# Supplementary material for: Molecular determinants of antagonist interactions with chemokine receptors CCR2 and CCR5
Source: bioRxiv. 2023 Nov 17:2023.11.15.567150. Preprint. [Version 1] doi: 10.1101/2023.11.15.567150 (PMC10680698; doi:10.1101/2023.11.15.567150)
Supplement: Supplementary file 1 [file media-1.pdf]

SUPPLEMENTARY ONLINE MATERIAL

Title: Molecular determinants of antagonist interactions with chemokine receptors CCR2 and CCR5

John R.D. Dawson[1], Grant M. Wadman[1], Penglie Zhang[2], Siyi Gu[1], Thomas Shroka[1], Leire Borrega-Roman[1], Catherina C. Salanga[1], Tracy M. Handel[1], Irina Kufareva[1]*

[1]Skaggs School of Pharmacy and Pharmaceutical Sciences, University of California San Diego, CA, USA

[2]ChemoCentryx, Mountain View, CA, USA

*Corresponding author: Irina Kufareva, Ph.D., Associate Adjunct Professor, Skaggs School of Pharmacy and Pharmaceutical Sciences, University of California San Diego, 9255 Pharmacy Lane, MC 0657, La Jolla, CA 92093. Phone: 858-822-4163. Email: ikufareva@ucsd.edu

Supplemental Figures and Legends

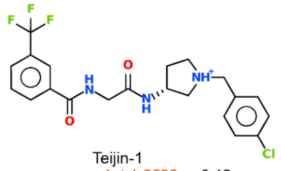
Teijin-1
pAct_hCCR2 6.43
pAct_hCCR5 0

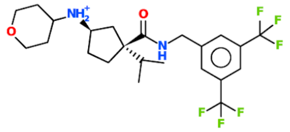
kothandaraman_34
pAct_hCCR2 8.167
pAct_hCCR5 0

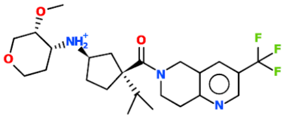
MK-0812-[*RR*]
pAct_hCCR2 8.5
pAct_hCCR5 0

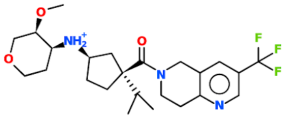
MK-0812-[*SS*]
pAct_hCCR2 8.301
pAct_hCCR5 0

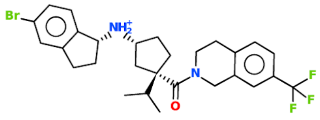
vilums15a_15a
pAct_hCCR2 8.62
pAct_hCCR5 0

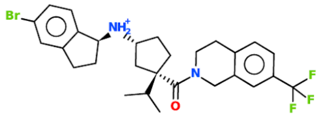
vilums15a_15b
pAct_hCCR2 7.62
pAct_hCCR5 0

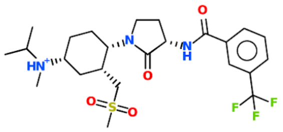
cherney14_2
pAct_hCCR2 8.738
pAct_hCCR5 5.786

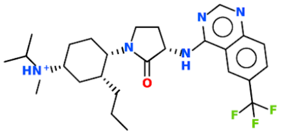
BMS-681
pAct_hCCR2 9.155
pAct_hCCR5 8.62

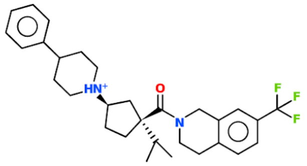
vilums15b_18
pAct_hCCR2 8.357
pAct_hCCR5 0

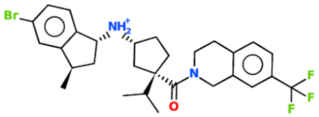
vilums15a_37b
pAct_hCCR2 8.337
pAct_hCCR5 0

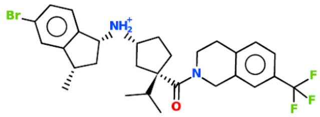
vilums15a_37a
pAct_hCCR2 8.77
pAct_hCCR5 0

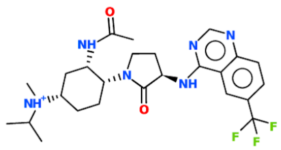
BMS-741672
pAct_hCCR2 8.917
pAct_hCCR5 6.106

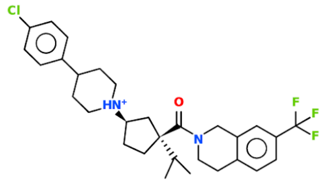
vilums15b_24
pAct_hCCR2 8.699
pAct_hCCR5 0

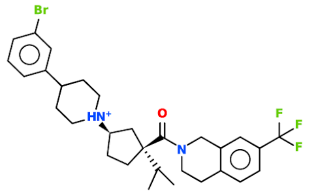
vilums15b_21
pAct_hCCR2 8.553
pAct_hCCR5 0

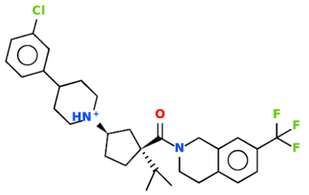
vilums15b_20
pAct_hCCR2 8.585
pAct_hCCR5 0

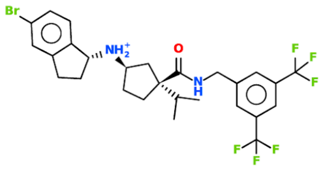
vilums13_22a
pAct_hCCR2 8.444
pAct_hCCR5 0

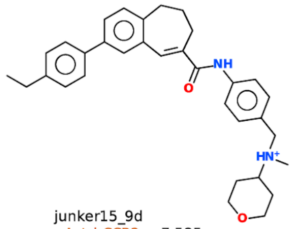
junker15_9d
pAct_hCCR2 7.585
pAct_hCCR5 7.398

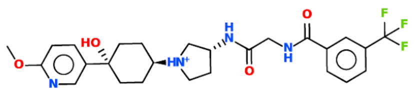
INCB-3284
pAct_hCCR2 8.418
pAct_hCCR5 4.699

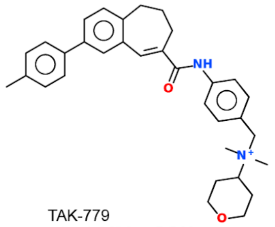
TAK-779
pAct_hCCR2 7.569
pAct_hCCR5 8.854

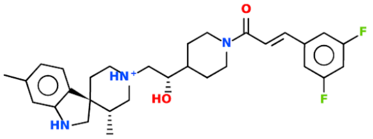
budzik08_56
pAct_hCCR2 8.699
pAct_hCCR5 0

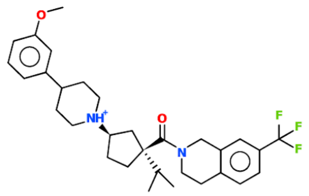
vilums15b_19
pAct_hCCR2 9.022
pAct_hCCR5 0

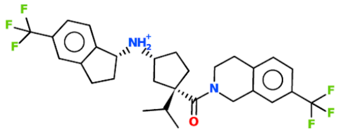
vilums15a_17a
pAct_hCCR2 7.886
pAct_hCCR5 0

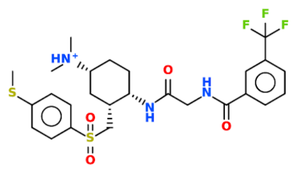
carter15_1
pAct_hCCR2 9.523
pAct_hCCR5 6

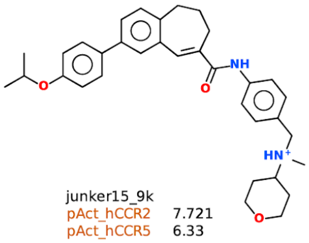
junker15_9k
pAct_hCCR2 7.721
pAct_hCCR5 6.33

Figure S1. Structures of public-domain orthosteric CCR2 and CCR5 antagonists used in this study.

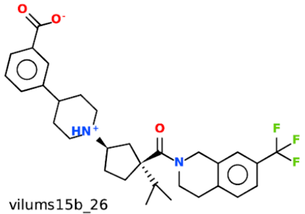

vilums15b_26
pAct_hCCR2 8.108
pAct_hCCR5 0

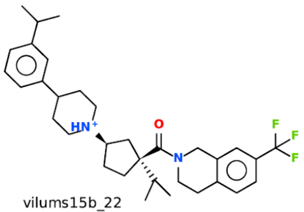

vilums15b_22
pAct_hCCR2 8.444
pAct_hCCR5 0

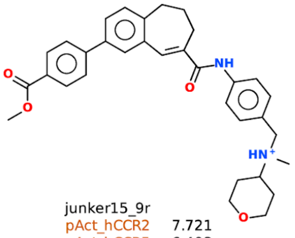

junker15_9r
pAct_hCCR2 7.721
pAct_hCCR5 6.403

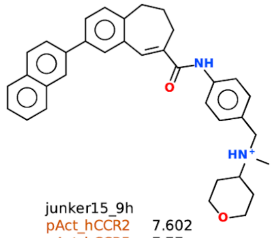

junker15_9h
pAct_hCCR2 7.602
pAct_hCCR5 7.77

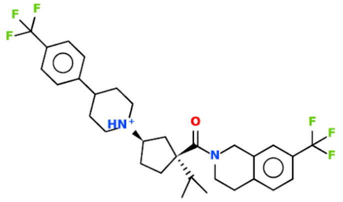

vilums15b_25
pAct_hCCR2 8.143
pAct_hCCR5 0

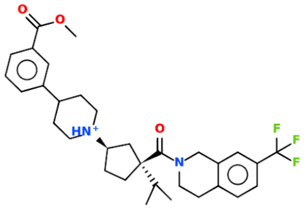

vilums15b_27
pAct_hCCR2 9.041
pAct_hCCR5 0

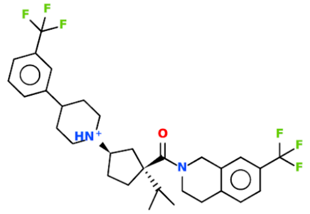

vilums15b_23
pAct_hCCR2 8.276
pAct_hCCR5 0

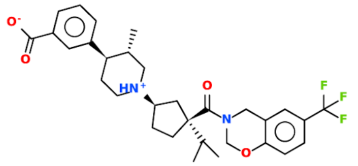

vilums15b_3d
pAct_hCCR2 7.959
pAct_hCCR5 0

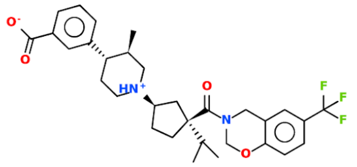

MK-0483
pAct_hCCR2 8.398
pAct_hCCR5 7.602

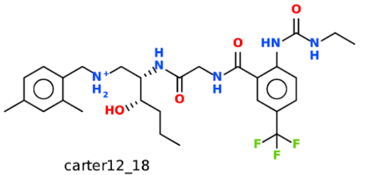

carter12_18
pAct_hCCR2 9.125
pAct_hCCR5 6.424

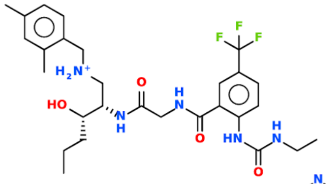

carter12_18
pAct_hCCR2 8.523
pAct_hCCR5 7.066

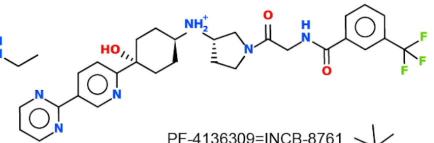

PF-4136309=INCB-8761
pAct_hCCR2 8.284
pAct_hCCR5 6

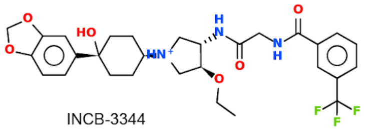

INCB-3344
pAct_hCCR2 8.292
pAct_hCCR5 0

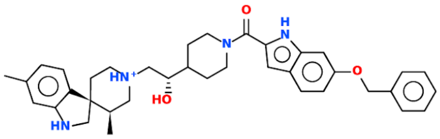

eidam10_21
pAct_hCCR2 7.038
pAct_hCCR5 0

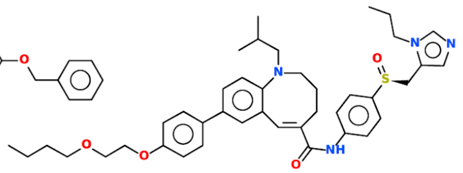

cenicriviroc
pAct_hCCR2 8.22
pAct_hCCR5 9.53

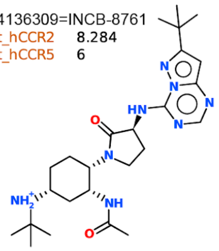

BMS-813160
pAct_hCCR2 8.2
pAct_hCCR5 8.4

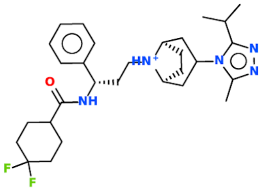

maraviroc
pAct_hCCR2 0
pAct_hCCR5 8.747

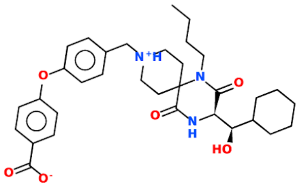

aplaviroc
pAct_hCCR2 0
pAct_hCCR5 8.033

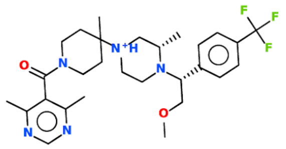

vicriviroc
pAct_hCCR2 0
pAct_hCCR5 7.9

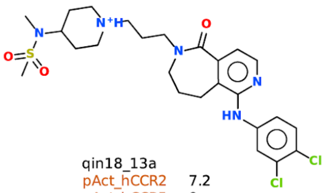

qin18_13a
pAct_hCCR2 7.2
pAct_hCCR5 0

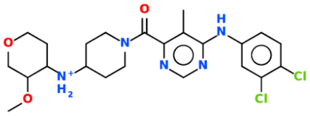

qin18_7
pAct_hCCR2 8.7
pAct_hCCR5 0

Figure S1 cntd. Structures of public-domain orthosteric CCR2 and CCR5 antagonists used in this study.

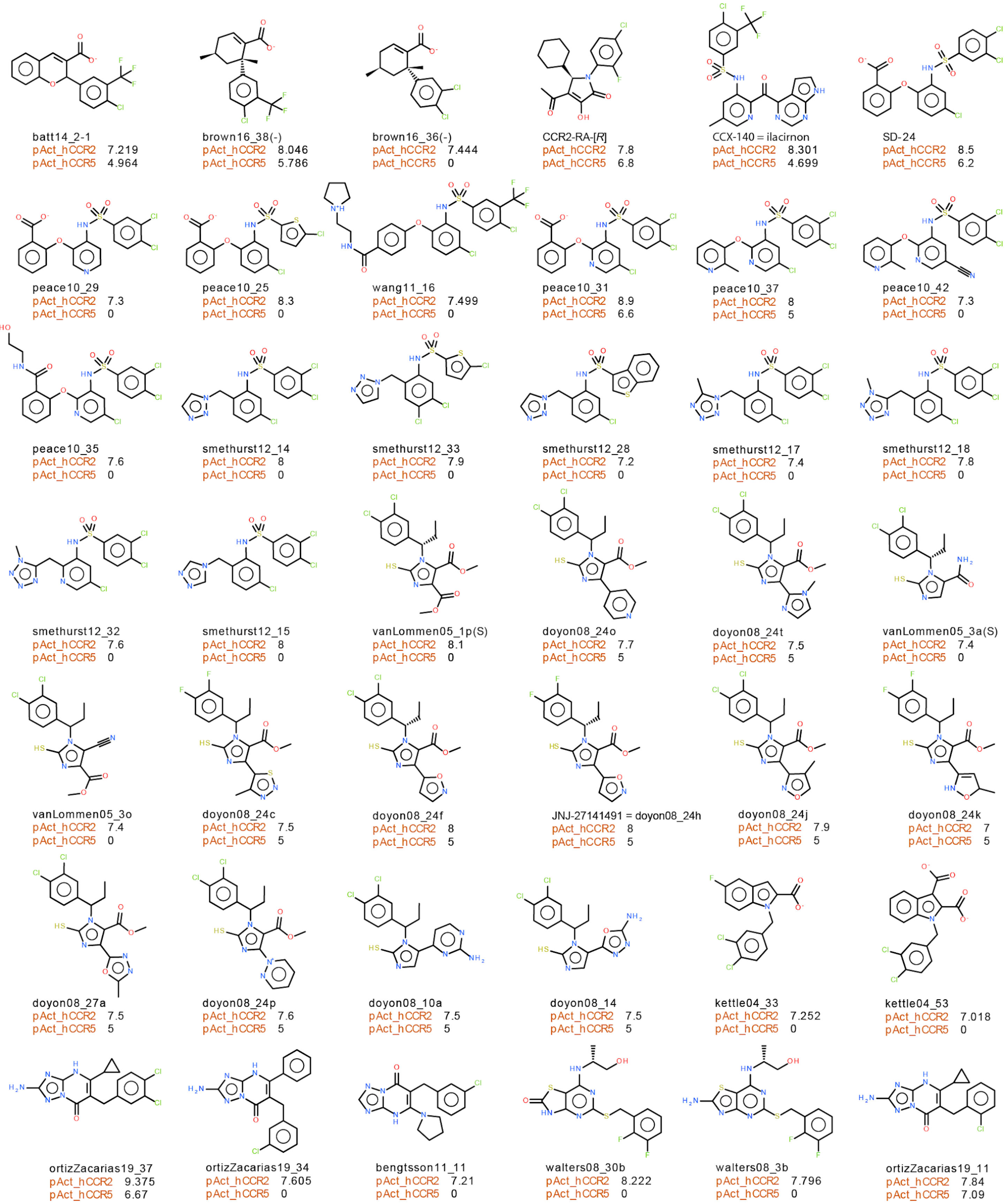

Figure S2. Structures of public-domain allosteric CCR2 antagonists used in this study.

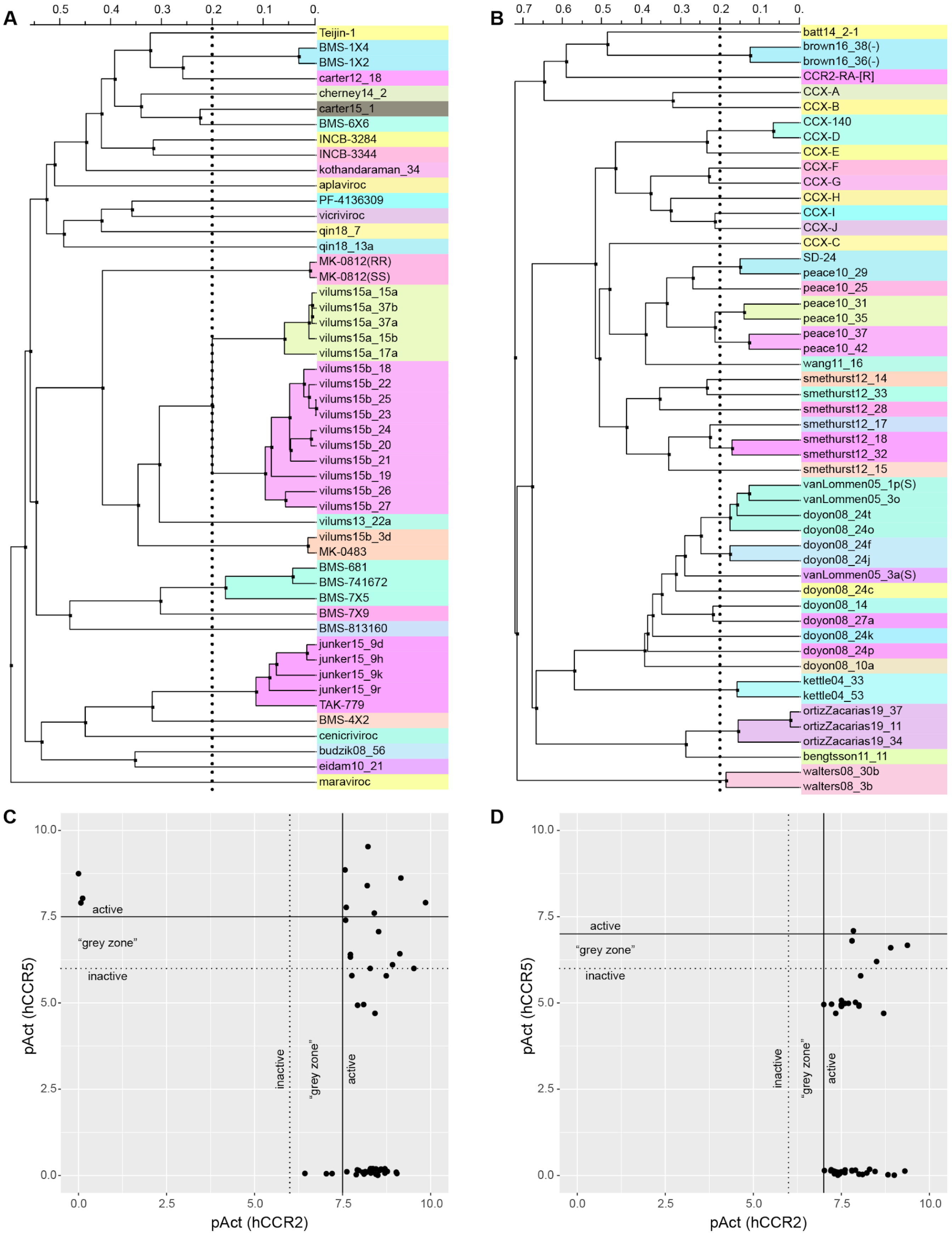

Figure S3. Compounds featured in this study are chemically and pharmacologically diverse.

(**A-B**) A hierarchical clustering dendrogram for the master library of 51 diverse orthosteric CCR2 and CCR5

antagonists (**A**) or the master library of 50 diverse allosteric antagonists (**B**) used in this study. Compounds were clustered by UPGMA based on Tanimoto distances of their chemical fingerprints as implemented in ICM. The dendrograms are colored by clusters emerging at the Tanimoto distance threshold of 0.2.
(C-D) Scatter plots of literature-derived inhibitory activities against human CCR2 vs human CCR5 for the 51 diverse orthosteric antagonists (**A**) or 50 allosteric antagonists (**B**) from the respective master libraries. Activities were expressed on a -log10 scale. Values close to zero indicate that the activity was not measurable or not available. Orthosteric antagonists with activities above 7.5 and allosteric compounds above 7 (solid lines) were considered active in all receiver operator characteristic (ROC) curves calculations. Orthosteric and allosteric compounds with activities below 6.5 or 6, respectively (dashed lines) were deemed inactive. Compounds within the "grey zone" between the solid and dashed lines were not regarded as active or inactive and were omitted when constructing ROC curves.

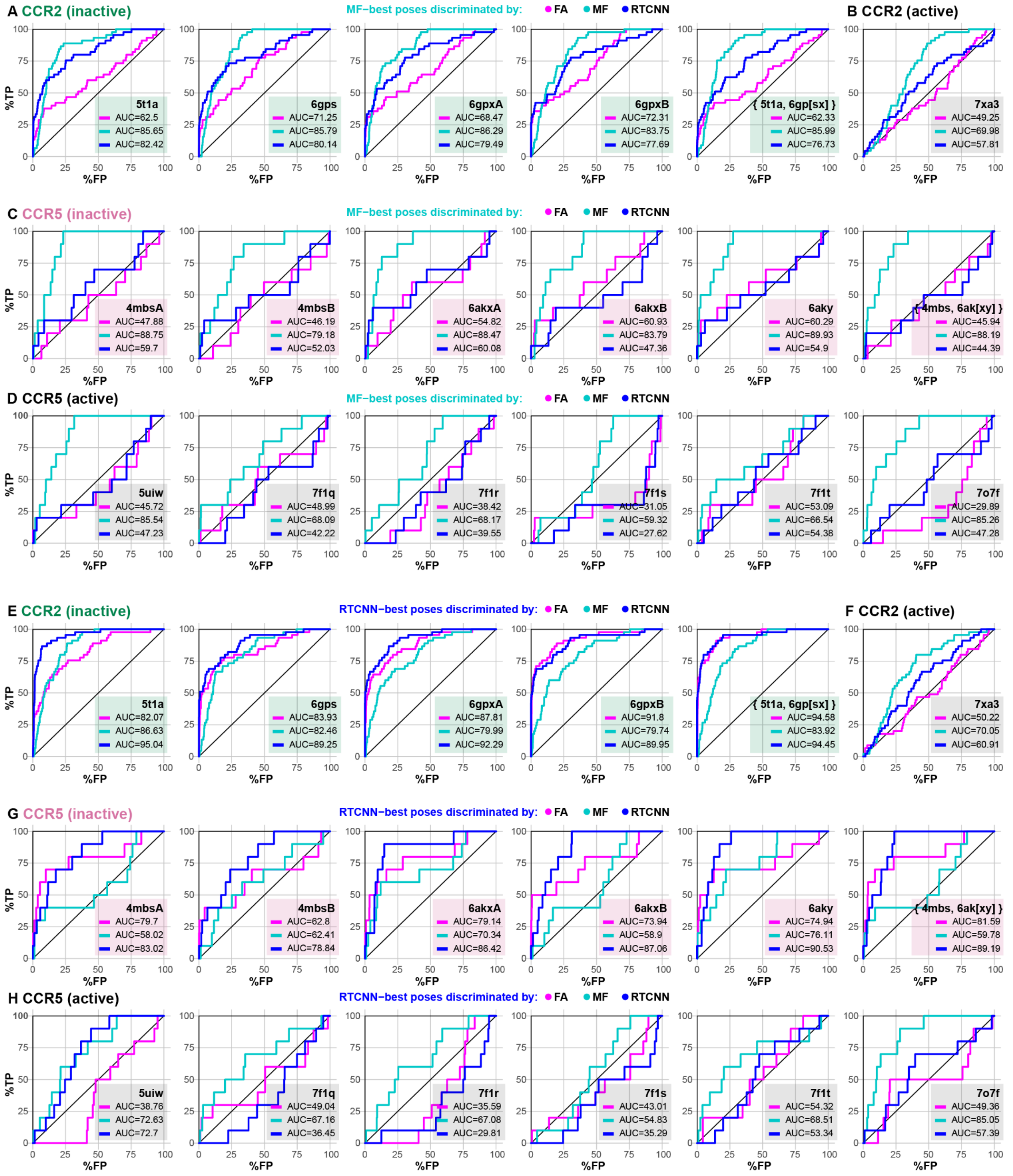

Figure S4. VLS recognition of known orthosteric antagonists by experimental structures of CCR2 and CCR5 when compound poses are selected using Mean-Force or RTCNN scoring.

(**A-B**) Receiver operator characteristic (ROC) curves obtained by VLS of a diverse library of 45 orthosteric CCR2 antagonists and a 50-fold larger set of property-matched DUDe decoys against the orthosteric pockets

in inactive (antagonist-bound) (**A**) and active (chemokine agonist-bound) (**B**) CCR2 structure(s). For each docked compound, a single predicted pose was selected using the mean-force (MF) scoring function [78] as implemented in ICM; compound ranking was performed using FA (magenta), MF (cyan), or RTCNN (blue).
(**C-D**) ROC curves obtained by VLS of a small diverse set 10 known CCR5 orthosteric antagonists and same inactives and decoys as in (**A-B**) against the orthosteric pockets in inactive (antagonist-bound) (**C**) and active (chemokine agonist-bound) (**D**) CCR5 structure(s). Pose selection and compound ranking were performed as in (**A-B**).
(**E-F**) ROC curves obtained by screening of the same library as in (**A-B**) against the same CCR2 structures, but with compound pose selection by RTCNN [79,80].
(**G-H**) ROC curves obtained by screening of the same library as in (**C-D**) against the same CCR5 structures, but with compound pose selection by RTCNN.

Figure S5. Characterization of WT CCR2, WT CCR5, and mutant CCR5 responses to CCL2 and CCL5 in a cAMP suppression assay.

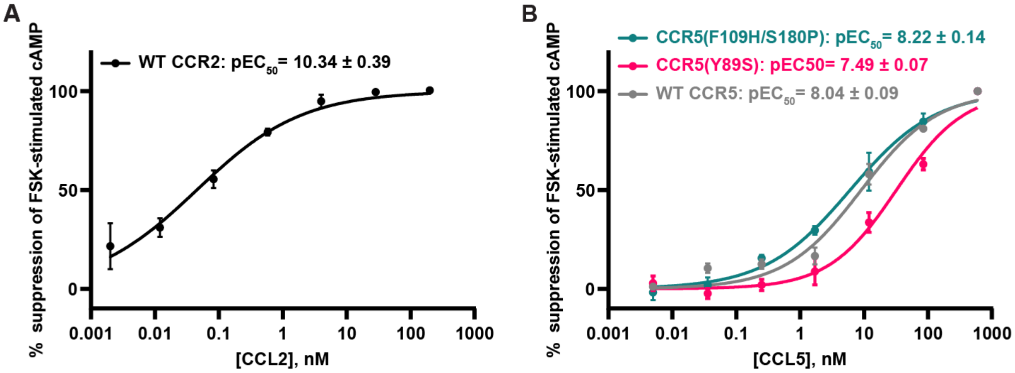

(**A-B**) Concentration-dependent suppression of forskolin-induced intracellular cAMP by CCL2 (**A**) and CCL5 (**B**) in HeLa cells transiently expressing WT CCR2 (**A**), WT CCR5 (**B**), CCR5(Y89S) (**B**), or CCR5(F109/S180P) (**B**). cAMP was measured via the BRET-based CAMYEL cAMP biosensor assay. Data within each experiment was normalized to 0%-100% as described in Methods. Error bars represent ±S.E.M. from n=3 independent experiments performed on different days, each with 2 technical replicates. Data was collated and fitted to a two-parameter nonlinear regression model (log[agonist] vs. normalized response with variable Hill slope) in GraphPad Prism.

Supplemental Tables

Supplemental Table 1. Overview of the chemical libraries of orthosteric and allosteric CCR2 antagonists used in this work.

**Orthosteric**				
**Chemotype**	**# CCR2 / dual [25] actives in the master library of 45* (pAct ≥ 7.5)**	**# cpds in the extended library**	**# CCR2 actives in the extended library (pAct cutoff)**	**References**
BI	1	200	83 (7.5)<br>34 (8.65)	[88,89,98]
BMS	12 / 3	530	330 (7.5)<br>65 (9.06)	[69,83–87]
GSK	1	NA	NA	[191]
INCB	3	NA	NA	[96,99,100]
MK	21 / 1	21	21 (7.5)	[26,51,90–92,94,95]
TK	7 / 3	NA	NA	[101,102]
**Allosteric**				
**Chemotype**	**# CCR2 actives in the master library of 50 (pAct ≥ 7)**	**# cpds in the extended library**	**# actives in the extended library (pAct cutoff)**	**References**
AZ	8	113	55 (7)	[120–123]
BMS	4	50	10 (7)	[129,130]
CCX	9	40	15 (7)	[131–133]
GSK	15	79	49 (7)	[128]
JNJ	13	93	30 (7)	[125–127]
UCB	1	52	7 (6.5)	[119,124]

*Not listed in this table are 3 orthosteric antagonists with pAct(CCR2) <7.5 and 3 CCR5-selective orthosteric antagonists, for the total of 45+3+3=51 orthosteric antagonists in the master orthosteric library.

Supplemental Table 2. Reagents, supplies, and equipment used in the experimental part of this work.

Reagent	Source	Product Number
**Chemicals, Reagents and Recombinant Proteins**		
PBS	Gibco	20020023
MEM	Gibco	32561-037
FBS	Avantor	97068-085
0.25% Trypsin + EDTA	Gibco	25200-072
TransIT-X2	Mirus Bio	MIR 6000
BSA	Sigma-Aldrich	9048-66-9
Glucose	VWR	97061-164
Coelenterazine-h	RPI Corp	C61500
IBMX	Sigma Aldrich	I5879
Forskolin	Sigma Aldrich	F3917
**PF-4136309**	MedChemExpress	HY-13245
**cherney08a_22** a.k.a. **BMS CCR2 22**	Tocris	3129
DMSO	Sigma-Aldrich	D2650
Q5 Site-Directed Mutagenesis Kit	New England Biolabs	E0554S
**Plasmids**		
V2-CAMYEL	[188,189]	
pcDNA3.1-Flag-hCCR2b	[186]	
pcDNA-Flag-hCCR5	Cloned in-house [185]	
pcDNA3.1-rGai3	Ghosh lab, UCSD	
**Primers (5'-3')**		
Y89S_fwd: TGGGCACATTCCGCAGCAGCC	Integrated DNA Technologies	
Y89S_rev: GAATGGCACAGTGAGCAGAAAG	Integrated DNA Technologies	
F109H_fwd: TGGCTTGTACAACATTGGCTTC	Integrated DNA Technologies	
F109H_rev: GTCAGGAGCTGACACATG	Integrated DNA Technologies	

S180P_fwd: TACTTGCTCCCCCCACTTCCCATAC	Integrated DNA Technologies	
S180P_rev: TAATGCAGTCCCTCCTTC	Integrated DNA Technologies	
**Other**		
TC-treated 6-well plate	Falcon	351146
96-well plate (white/clear bottom)	Greiner	655098
White backing tape	PerkinElmer	6005199
Spark Microplate Reader	TECAN	

Supplemental Data 1. Orthosteric complexes featured in this work (3D coordinates in the PDB format)

Supplemental Data 2. Allosteric complexes featured in this work (3D coordinates in the PDB format)
